# Characteristics of hemoglobin distributions in preschool children and non-pregnant women of reproductive age and their implications for establishing quality control criteria for hemoglobin data in field surveys: evidence from 483 surveys conducted in refugee settings worldwide

**DOI:** 10.1186/s12963-023-00315-9

**Published:** 2023-11-09

**Authors:** Oleg Bilukha, Behzad Kianian, Kaitlyn L. I. Samson

**Affiliations:** 1 Emergency Response and Recovery Branch, Division of Global Health Protection, Global Health Center, Centers for Disease Control and Prevention, 1600 Clifton Road NE, Atlanta, GA 30333 USA; 2 Action Against Hunger Canada, Toronto, ON Canada

**Keywords:** Hemoglobin, Anemia, Assessment, Measurement, Surveillance, Surveys, Data quality, Nutrition

## Abstract

**Background:**

Currently, there is a lack of clear guidance on hemoglobin (Hb) data quality parameters and plausible flagging ranges for population-representative surveys. There is a need to determine which properties of Hb data indicate lower data quality and increased measurement error and which represent intrinsic statistical properties of Hb distributions rather than quality problems.

**Methods:**

We explored statistical characteristics of Hb distributions and plausible exclusion ranges in population-representative surveys of non-pregnant women of reproductive age (WRA) (15–49 years, *n* = 401 surveys) and children (6–59 months, *n* = 461 surveys) conducted in refugee settings by the United Nations High Commissioner for Refugees (UNHCR). Hb distribution characteristics [standard deviation (SD), skewness and kurtosis] were compared to those from Demographic and Health Surveys (DHS).

**Results:**

Overall, 0.08% of child and 0.14% of WRA Hb values were outside of the previously proposed 4.0–18.0 g/dL plausible range. Surveys conducted in Uganda tended to have unusually high SD compared with surveys from other settings, possibly an indication of problematic measurement quality. We therefore used summary results on SD, skewness and kurtosis excluding surveys from Uganda when comparing with DHS results or proposing plausible ranges. Both WRA and child Hb distributions tended to be left-skewed and had excess positive kurtosis. Mean survey-level SD was greater, mean skewness more negative, and mean kurtosis more positive in WRA surveys compared to child surveys. All these findings were broadly similar to those from DHS surveys. Mean SD in DHS surveys was higher than that in our data for both children (1.48 vs*.* 1.34) and WRA (1.58 vs*.* 1.43).

**Conclusions:**

We observed several statistical characteristics of Hb distributions that may not necessarily be indicative of data quality problems and bear strong similarities with the characteristics found in DHS surveys. Hb distributions tended to be negatively skewed and positively kurtotic, and SD in many surveys exceeded 1.5 (previously proposed upper plausible range). Based on our empirical evidence, surveys with skewness above + 0.2 and kurtosis below -0.5 or Hb SD outside the range of 1.1–1.55 g/dL for children (6–59 mo) or 1.1–1.65 g/dL for non-pregnant WRA (15–49 y) may require further quality investigation.

## Background

Anemia, as determined by hemoglobin (Hb) concentration, is a problem of significant public health concern. In 2019, the global estimated prevalence of anemia was 30% among women of reproductive age (15–49 years, WRA) and 40% among children aged 6–59 months, with similar estimates found among displaced women and children [1, 2]. Progress toward international reduction targets has stagnated, and the World Health Organization (WHO) has called for a better understanding of the burden through more reliable assessments of Hb concentration [1, 3]. In field settings, portable photometric point-of-care analyzers (e.g., HemoCue devices) represent the current standard of care for hemoglobin determination [4, 5]. However, these devices are subject to numerous preanalytic and analytic factors that introduce bias and imprecision [4, 6–8].

In order to assess the anemia burden, there is a need for valid and robust Hb estimates from population-representative surveys. To date, limited research has explored Hb distributions in nationally representative population-based surveys [9, 10], and there is a further dearth of research among displaced persons. Currently, no clear guidance exists for the quality control of Hb data in the postanalytic phase [4, 8, 11]. This is especially true for population-representative surveys, where two important metrics of measurement quality, the standard deviation (SD) of Hb distribution and the percentage of implausible extreme Hb values (flags), lack definitive guidance on acceptable ranges. Indeed, commonly cited values for acceptable SDs of Hb distribution (1.1–1.5 g/dL range), as determined by the HemoCue system in cross sectional surveys, are based on the authors’ empirical experience (with no supporting data presented) [12]. Multiple ranges for minimum and maximum plausible Hb values for non-pregnant women and children have been described and used in recent literature without further justification, for example, 2.0 g/dL to 30.0 g/dL [7], 2.5 g/dL to 20.0 g/dL [1], 4.0 to 18.0 g/dL [12], and 4.0 g/dL to 21.0 g/dL [13].

The consideration of these two metrics is important as measurement error can lead to increased dispersion (SD) of the distribution and, thus, the overestimation of the proportion of the population with anemia [9]. A 2017 report exploring the quality of Hb data in Demographic and Health Surveys (herein referred to for brevity as the “DHS Report”) found important differences between Hb distributions in WRA and children. This report analyzed various parameters of Hb distributions in 80 DHS surveys from multiple countries conducted between 2000 and 2016. All 80 of these surveys had child Hb data and 65 had WRA Hb data. The authors found that Hb SDs above 1.5 g/dL are quite common for both children (46% of surveys, excluding implausible values outside of the 4.0 to 18.0 g/dL range) and WRA (71% of surveys, excluding implausible values in the same range) [9]. Important peculiarities in Hb distribution shape were also noted for both WRA and children: Hb distributions were left-skewed and positively kurtotic. Both SD, left skew and positive kurtosis tended to be larger in WRA Hb distributions than in child Hb distributions [9]. Following the exclusion of implausible values, the overall mean SDs for children and WRA across all analyzed surveys were 1.48 g/dL and 1.58 g/dL, respectively—suggesting a potential need to revisit the commonly cited upper acceptable SD value of 1.5 g/dL [9, 12].

Given the lack of clear guidance on Hb data quality parameters and acceptable ranges, there remains a need to determine which properties of Hb data indicate lower data quality and increased measurement error and which represent intrinsic statistical properties of population Hb distributions and not necessarily quality problems [8–10]. This study aimed to explore statistical characteristics of Hb distributions, plausible exclusion ranges, and the probability of observing extreme outliers using data from population-representative surveys of non-pregnant women aged 15–49 years and children aged 6–59 months in refugee settings. We also compared these distribution characteristics with those found in large national surveys as described in the DHS report [9].

## Methods

Surveys were conducted in refugee and emergency settings from 2013 to 2021 by the United Nations High Commissioner for Refugees (UNHCR) and its implementing partners. Questionnaires, tools, and sampling methods were based on the UNHCR Standardized Expanded Nutrition Survey (SENS) guidelines; survey designs consisted of two-stage cluster samples, systematic or simple random samples, or exhaustive samples [14, 15].

For surveys that included the anemia module, Hb measurements were taken using the HemoCue Hb 301 Analyzer [5, 16]. Per the manufacturer’s specification, values outside of 0 to 25.6 g/dL were considered invalid for this analysis. Altitude-adjusted Hb values were used in the provided data, which followed guidelines provided by UNHCR SENS [5]. Anemia was classified as < 11.0 g/dL for children aged 6 to 59 months and < 12.0 g/dL for non-pregnant women aged 15–49 years, as described by WHO guidelines [5, 11]. Surveys were included in the analysis if Hb measurements were taken and provided for children aged 6 to 59 months, non-pregnant women aged 15 to 49 years, or both groups. We limited our analysis to surveys with at least 30% valid Hb values to include surveys that may have conducted sub-sampling of respondents for anemia analyses. According to SENS guidelines, either all or half of the households selected in the survey are used to conduct Hb measurements in eligible household members [5]. Within each survey, we excluded duplicated observations prior to analysis.

First, to broadly describe the dataset, we classified the included surveys by country and by year and described the total number of children and non-pregnant WRA, the number of surveys, and the number of unique sites in each of these groupings. One of the goals of the present analysis was to assess the frequency of extreme recorded Hb measurements. To this end, we calculated the number of extreme values for each survey and, across all surveys, the overall percentage of valid Hb observations below 4.0, 5.0, and 6.0 g/dL and above 16.0, 17.0, and 18.0 g/dL. Another goal was to assess the shape of Hb distributions. Thus, for each survey, the mean, median, standard deviation (SD), skewness, and kurtosis of Hb and anemia prevalence were estimated based on Hb values in the range of 4.0 to 18.0 g/dL, following the existing literature on ranges [12]. For each of these summary statistics, we then calculated the mean, SD, and various quantiles (minimum, 2.5%, 10%, 25%, 50%, 75%, 90%, 97.5%, maximum). We then produced histograms of the distributions of SD, kurtosis, and skewness across surveys for both WRA and children. Among both WRA and children, we examined the relationship between Hb survey-level means and SDs with scatter plots and estimates of the Spearman correlation coefficient. For all analyses including kurtosis, we presented excess kurtosis (subtracting 3 from kurtosis), which is centered at zero under a normal distribution.

To determine whether Hb has different distributional properties in WRA versus children, among the surveys where both children and WRA with Hb data were measured, we summarized and compared the average SDs, skewness, and kurtosis of Hb measurements. Among these paired observations, we also plotted SDs, kurtosis, and skewness estimates for WRA and children to better understand their relationship within the same survey. Finally, we compared several key results from our analysis with those from the *DHS Methodological Reports No.18* which analyzed Hb distributions in national-level DHS surveys [9].

Data aggregation and cleaning were done in SAS and R 4.0.3 [17, 18]. Survey-level summary statistics were calculated using R version 4.0.3; skewness and kurtosis were estimated using the *G*_*1*_ and *G*_*2*_ methods, respectively [19, 20]. Plots and quantiles were performed in Stata version 15 [21]. This study constituted a secondary analysis of routinely collected de-identified programmatic data, it was reviewed by the Centers for Disease Control and Prevention (CDC) and determined to be conducted consistent with applicable federal law and CDC policy (45 C.F.R. part 46, 21 C.F.R. part 56; 42 U.S.C. Section 241(d); 5 U.S.C. Section 552a; 44 U.S.C. Section 3501 et seq.).

## Results

Data for these analyses come from 483 surveys conducted by UNHCR in refugee and emergency settings from 2013–2021. There were no surveys conducted in 2020 due to the COVID-19 pandemic. In total, 461 surveys included valid Hb data for children aged 6–59 months, 401 surveys included valid Hb data for non-pregnant WRA aged 15–49 years, and 379 surveys included data for both WRA and children (Table 1). Median sample size of child surveys was 400 (5^th^ percentile: 175; 95^th^ percentile: 649), median sample size of WRA surveys was 226 (5^th^ percentile: 99; 95^th^ percentile: 406). Overall, 187,641 child and 93,731 WRA records were included in the analysis. Data were available from refugee settings located in 26 countries across four UNICEF regions [22], with Ethiopia, Chad and Uganda having the largest number of surveys. Full information on the numbers of surveys, unique cites and sample sizes per country and region is presented in Table 1.

**Table 1:** Included hemoglobin surveys, by country, and year

	Children 6–59 months			Non-pregnant women 15–49 years			Overlapping Surveys			
	Number of surveys	Number of unique sites	Number of children	Number of surveys	Number of unique sites	Number of WRA	Number of surveys assessing both WRA and children	Number of unique sites	Number of children	Number of WRA
Total	461	185	187,641	401	177	93,731	379	172	160,002	88,259
Middle East and North Africa	5	5	1706	3	3	1320	3	3	1211	1320
Jordan	2	2	794	2	2	918	2	2	794	918
Yemen	3	3	912	1	1	402	1	1	417	402
South Asia	9	4	3992	7	3	2130	5	3	1634	1497
Bangladesh	6	3	3104	4	2	1427	2	2	746	794
Nepal	3	1	888	3	1	703	3	1	888	703
Eastern and Southern Africa	314	112	121,346	254	105	55,674	244	104	98,899	53,104
Botswana	–	–	–	1	1	146	–	–	–	–
Burundi	4	4	1069	4	4	527	4	4	1069	527
Djibouti	5	3	1400	4	2	607	4	2	1235	607
Eritrea	2	1	649	1	1	97	1	1	326	97
Ethiopia	104	27	38,016	92	27	15,551	90	27	32,573	15,152
Kenya	32	7	18,443	37	7	10,737	31	7	17,777	8854
Malawi	5	4	1840	5	4	1190	5	4	1840	1190
Mozambique	2	2	225	1	1	88	1	1	184	88
Rwanda	36	6	11,967	6	6	2875	6	6	2125	2875
South Sudan	37	10	17,397	35	10	8099	35	10	16,428	8099
Sudan	13	10	4252	13	10	2239	13	10	4252	2239
Uganda	56	32	16,888	39	26	9920	38	26	13,241	9778
United Republic of Tanzania	16	4	8821	14	4	3249	14	4	7470	3249
Zambia	2	2	379	2	2	349	2	2	379	349
West and Central Africa	133	64	60,597	137	66	34,607	127	62	58,258	32,338
Burkina Faso	5	2	693	9	4	1141	5	2	693	773
Cameroon	11	11	5109	10	10	2446	10	10	4730	2446
Chad	87	29	45,009	90	29	24,056	87	29	45,009	23,632
Congo	5	5	1171	5	5	1018	5	5	1171	1018
Democratic Republic of the Congo	8	7	2616	8	8	2777	6	6	1905	1883
Liberia	7	4	2148	7	4	905	7	4	2148	905
Mauritania	5	1	2207	3	1	1623	2	1	958	1040
Niger	5	5	1644	5	5	641	5	5	1644	641
Total, by year										
2013	53	53	22,461	49	49	11,526	45	45	19,192	10,622
2014	55	55	22,412	40	40	9390	35	35	15,498	8473
2015	62	62	20,220	53	53	10,683	44	44	16,679	8325
2016	66	66	29,722	59	59	13,922	59	59	27,383	13,922
2017	85	85	35,754	76	76	17,420	74	74	30,610	17,021
2018	38	37	15,095	28	27	5265	28	27	11,394	5265
2019	43	43	16,884	37	37	9523	35	35	14,153	8629
2021	59	59	25,093	59	59	16,002	59	59	25,093	16,002

*WRA* Women of reproductive age

Table 2 describes the Hb outlier values for WRA and children across all surveys. Approximately 0.08% of all child and 0.14% of WRA records were outside of the 4.0–18.0 g/dL range. Overall, only 2.2% of surveys in children and 3.2% of surveys in WRA had more than 1% of outliers outside of the 4.0–18.0 g/dL range. On the other hand, 88.3% of child and 90.3% of WRA surveys had no Hb values outside of the 4.0–18.0 g/dL range.

**Table 2:** Hemoglobin outlier values for non-pregnant women and children across all surveys, by exclusion cutoff

	Hemoglobin Outlier Cut-offs						
	< 4.0g/dL	< 5.0 g/dL	< 6.0 g/dL	> 16.0 g/dL	> 17.0 g/dL	> 18.0 g/dL	< 4.0g/dL or > 18g/dL
Child Records (*n* = 187,641)							
Percentage of all children in a given outlier range	0.04%	0.08%	0.22%	0.07%	0.04%	0.03%	0.08%
Child Surveys (*n* = 461)							
Percentage of surveys with no outliers	93.7%	83.7%	63.1%	84.2%	92.0%	94.1%	88.3%
Percentage of surveys with 0–1% outliers	4.6%	14.1%	29.9%	14.5%	7.2%	5.4%	9.5%
Percentage of surveys with > 1% outliers	1.7%	2.2%	6.9%	1.3%	0.9%	0.4%	2.2%
WRA Records (*n* = 93,731)							
Percentage of all WRA in a given outlier range	0.04%	0.07%	0.13%	1.36%	0.19%	0.09%	0.14%
WRA Surveys (*n* = 401)							
Percentage of surveys with no outliers	97.3%	92.0%	82.0%	44.1%	78.8%	91.3%	90.3%
Percentage of surveys with 0–1% outliers	2.0%	7.0%	15.2%	26.2%	16.7%	6.7%	6.5%
Percentage of surveys with > 1% outliers	0.8%	1.0%	2.7%	29.7%	4.5%	2.0%	3.2%

*WRA* Women of reproductive age

Overall, the average survey-level mean Hb in WRA was much higher than in children (12.6 g/dL vs. 11.1 g/dL) (Table 3). Consequently, the mean prevalence of total anemia was higher (43.5%) in child surveys than in WRA surveys (30.2%). Mean survey-level SD was greater (1.47 vs. 1.39), mean skewness was more negative (− 0.53 vs. − 0.37), and mean kurtosis was more positive (1.15 vs. 0.54) in WRA surveys compared with child surveys. On further investigation, we noted that a large proportion of the surveys with very large (above the 90^th^ percentile across all surveys) SD both in children and WRA were from Uganda: 36 out of 46 surveys in children and 20 out of 40 in WRA. We therefore repeated the analysis of SD in Table 3 excluding all Uganda surveys in children and WRA. In this analysis, the 90^th^ percentile of SD across all remaining surveys in children decreased from 1.67 to 1.53, and in WRA from 1.74 to 1.66. We also include in Table 3 skewness and kurtosis analysis excluding Uganda surveys, which did not meaningfully differ before and after exclusion.

**Table 3:** Survey-level hemoglobin distribution parameters and prevalence of total anemia in children and women

	Mean Hb^a^	Median Hb^a^	% Total Anemia	SD^a^	SD excluding Uganda^b^	Skewness	Skewness excluding Uganda^b^	Kurtosis	Kurtosis excluding Uganda^b^
*Child surveys* (*n* = 461)									
Mean	11.1	11.2	43.5	1.39	1.34	− 0.37	− 0.38	0.54	0.55
SD	0.50	0.49	13.6	0.21	0.15	0.26	0.23	0.67	0.66
Maximum	12.5	12.5	81.7	2.37	1.84	1.25	0.78	5.03	5.03
P97.5	12.1	12.1	72.1	1.93	1.67	0.10	0.05	2.18	2.17
P90	11.7	11.8	61.3	1.67	1.53	− 0.09	− 0.09	1.37	1.37
P75	11.4	11.5	52.4	1.48	1.44	− 0.24	− 0.24	0.84	0.82
P50	11.1	11.2	42.9	1.34	1.32	− 0.38	− 0.38	0.42	0.44
P25	10.8	10.9	33.3	1.25	1.23	− 0.51	− 0.51	0.10	0.10
P10	10.4	10.5	26.6	1.18	1.17	− 0.67	− 0.67	− 0.14	− 0.10
P2.5	10.1	10.1	18.6	1.09	1.09	− 0.81	− 0.83	− 0.34	− 0.27
Minimum	9.6	9.7	12.2	1.01	1.01	− 1.32	− 1.32	− 1.05	− 0.51
*WRA Surveys* (*n* = 401)									
Mean	12.6	12.7	30.2	1.47	1.43	− 0.53	− 0.55	1.15	1.22
SD	0.57	0.56	13.2	0.23	0.18	0.36	0.36	1.17	1.18
Maximum	13.9	13.9	74.5	2.56	2.01	0.77	0.77	8.40	8.40
P97.5	13.7	13.8	58.0	2.11	1.79	0.18	0.17	3.87	4.05
P90	13.4	13.5	47.6	1.74	1.66	− 0.08	− 0.09	2.83	2.87
P75	13.0	13.0	38.4	1.57	1.54	− 0.28	− 0.32	1.62	1.68
P50	12.6	12.7	29.2	1.44	1.42	− 0.53	− 0.55	0.89	0.93
P25	12.2	12.3	20.9	1.31	1.29	− 0.76	− 0.78	0.38	0.43
P10	11.9	12.0	13.2	1.20	1.20	− 0.98	− 0.99	− 0.04	0.06
P2.5	11.6	11.6	7.5	1.13	1.12	− 1.32	− 1.37	− 0.38	− 0.24
Minimum	10.6	10.45	2.8	1.00	1.00	− 1.87	− 1.87	− 0.81	− 0.81

Data exclude Hb values < 4.0 g/dL and > 18.0 g/dL. *Hb* Hemoglobin; *P* Percentile; *SD* Standard deviation; *WRA* Women of reproductive age

^a^Values are in g/dL

^b^Data exclude surveys conducted in Uganda (*n* = 56 child surveys and *n* = 39 WRA surveys)

**Fig. 1:**
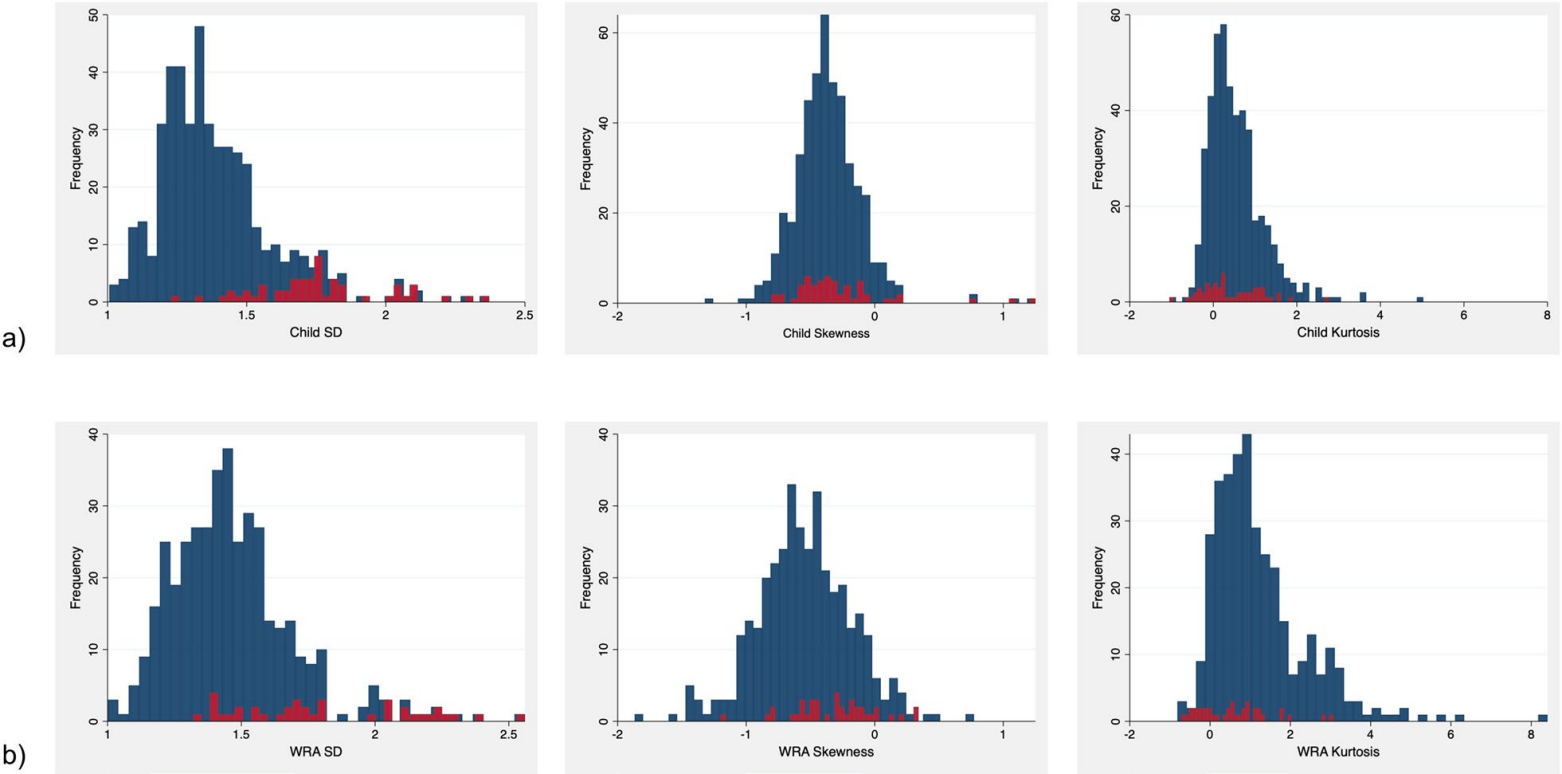
Survey-level distributions of standard deviation, skewness, and kurtosis among 461 child surveys and 401 WRA surveys. Distributions of survey-level standard deviation, skewness, and kurtosis among **a** children (6–59 mo) and **b** non-pregnant WRA (15–49 y) using a Hb exclusion range of 4.0–18.0 g/dL. Red bars indicate the distribution of SD, skewness, and kurtosis for surveys conducted in Uganda (*n* = 56 child surveys and *n* = 39 WRA surveys). *Hb* Hemoglobin; *SD* standard deviation; *WRA* Women of reproductive age

**Fig. 2:**
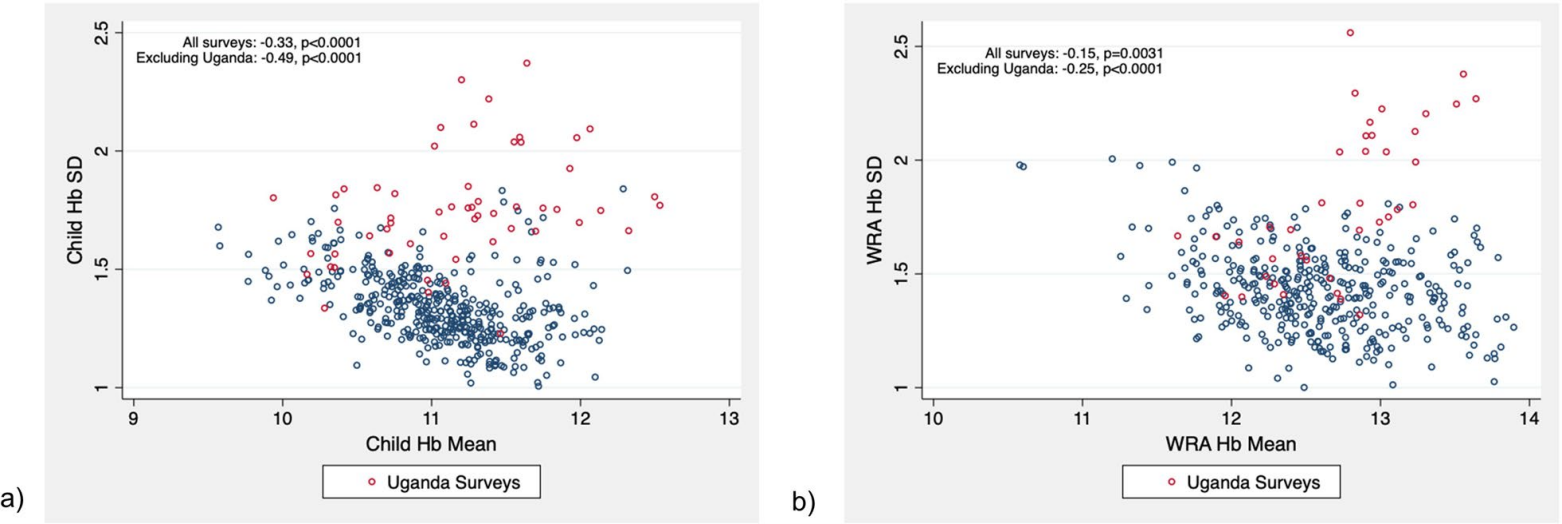
Correlation between survey-level hemoglobin means and standard deviations in 461 child surveys and 401 women surveys. Correlation of **a** child (6–59 mo) survey-level Hb SD and Hb means and **b** non-pregnant WRA (15–49 y) survey-level Hb SD and Hb means using a Hb exclusion range of 4.0–18.0 g/dL. Red circles represent data from surveys conducted in Uganda (*n* = 56 child surveys and *n* = 39 WRA surveys). Spearman’s rho was calculated both including and excluding Uganda data. *Hb* Hemoglobin; *SD* Standard deviation; *WRA* Women of reproductive age

**Fig. 3:**
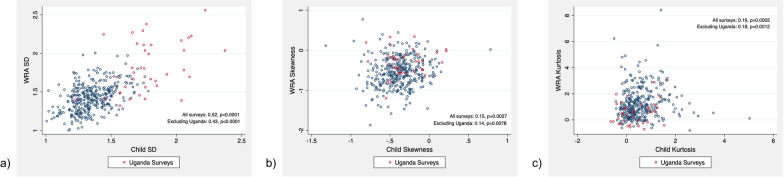
Correlation of survey-level standard deviations, skew, and kurtosis in the 379 overlapping surveys. Legend: Correlation of **a** children and WRA survey-level SDs, **b** children and WRA survey-level skewness, and **c** children and WRA survey-level kurtosis using a Hb exclusion range of 4.0–18.0 g/dL. Red circles represent data from surveys conducted in Uganda (*n* = 38 overlapping surveys). Spearman’s rho was calculated both including and excluding Uganda data. *Hb* Hemoglobin; *SD* Standard deviation; *WRA* Women of reproductive age

Survey-level distributions of standard deviation, skewness, and kurtosis are presented in Fig. 1, with surveys from Uganda marked in red for visibility. For both children and WRA, there was a negative survey-level correlation between the mean and SD of Hb (Spearman’s rho = -0.33, *p* < 0.0001 and Spearman’s rho = -0.15, *p* = 0.003, respectively) (Fig. 2). After excluding Uganda surveys correlations for both children and WRA increased to -0.49 and -0.25, respectively. Among surveys that measured Hb in both children and WRA, both mean SD, skewness, and kurtosis were significantly different (*p* < 0.0001) (Table 4). The difference followed the same direction as described for all surveys: WRA had higher SD, more positive kurtosis, and more negative skewness. Hb SD in WRA and children in the same surveys were highly correlated (Spearman’s rho = 0.52, *p* < 0.0001), while correlations of skewness and kurtosis between WRA and children were much smaller (Spearman’s rho = 0.15, *p* = 0.003, and Spearman’s rho = 0.19, *p* = 0.0002, respectively) (Fig. 3). These correlations did not change meaningfully after excluding Uganda surveys.

**Table 4:** Comparison of survey-level standard deviations, skewness, and kurtosis of hemoglobin distributions in the 379 overlapping surveys

	Mean SD Children (SD)	Mean SD WRA (SD)	Difference (95% CI)	Mean Skew Children (SD)	Mean Skew WRA (SD)	Difference (95% CI)	Mean Kurtosis Children (SD)	Mean Kurtosis WRA (SD)	Difference (95% CI)
Including Uganda surveys	1.39 (0.20)	1.47 (0.23)	− 0.07 (− 0.09, − 0.05)*	− 0.37 (0.23)	− 0.52 (0.37)	0.15 (0.11, 0.19)*	0.53 (0.66)	1.16 (1.17)	− 0.63 (− 0.76, − 0.50)*
Excluding Uganda surveys^a^	1.35 (0.14)	1.43 (0.18)	− 0.08 (− 0.09, − 0.06)*	− 0.38 (0.23)	− 0.55 (0.37)	0.17 (0.12, 0.21)*	0.55 (0.66)	1.22 (1.18)	− 0.68 (− 0.82, − 0.54)*

Data exclude Hb values < 4.0 g/dL and > 18.0 g/dL. *CI* Confidence interval; *Hb* Hemoglobin; *SD* Standard deviation; *WRA* Women of reproductive age

^a^Calculations exclude data from *n* = 38 overlapping surveys conducted in Uganda

^*^P < 0.0001

Table 5 presents the comparison of SD, skewness and kurtosis in our analysis (excluding Uganda surveys as described above) to those from the DHS report. In comparison with DHS surveys, UNHCR surveys had lower mean survey-level SD for both WRA (1.43 vs. 1.58) and children (1.34 vs. 1.48), as well as a lower percentage of surveys with SD > 1.5 for both WRA and children. Mean skew for children was similar between UNHCR and DHS surveys (− 0.38 vs. − 0.39) while it was slightly more negative among DHS surveys for WRA (− 0.61 vs*.* − 0.55). Mean kurtosis was similar between UNHCR and DHS surveys for both children (0.55 vs. 0.54) and WRA (1.22 vs. 1.25).

**Table 5:** Comparison of survey-level standard deviations, skewness, and kurtosis of hemoglobin distributions between UNHCR and DHS Surveys

	Child Surveys		WRA Surveys	
	UNHCR^a^ *n* = 405	DHS^b^ *n* = 80	UNHCR^a^ *n* = 362	DHS^b^ *n* = 65
Mean SD	1.34	1.48	1.43	1.58
% of surveys with SD < 1.1	4.0%	0.0%	1.9%	0.0%
% of surveys SD > 1.5	13.1%	46.3%	32.9%	70.8%
Mean Skew	-0.38	-0.39	-0.55	-0.61
% of surveys with skew < -0.5	27.4%	20.0%	55.8%	75.4%
% of surveys with skew > 0.5	0.2%	0.0%	0.3%	0.0%
Mean Kurtosis	0.55	0.54	1.22	1.25
% surveys with excess kurtosis > 1	18.5%	6.3%	47.0%	61.5%

Data exclude Hb values < 4.0 g/dL and > 18.0 g/dL. *DHS* Demographic and Health Surveys; *Hb* Hemoglobin; *SD* Standard deviation; *UNHCR* United Nations High Commissioner for Refugees; *WRA* Women of reproductive age

^a^Data exclude surveys conducted in Uganda (*n* = 56 child surveys and *n* = 39 WRA surveys)

^b^Data were obtained from *DHS Methodological Reports No. 18* [9]

## Discussion

Our analysis, which was based on a large number of recent field surveys conducted in refugee settings worldwide that followed the SENS survey guidelines and used standard equipment and procedures for Hb collection, produced several remarkable findings that have important implications for formulating criteria for quality assessment of Hb survey data in the post-data collection phase. Below, we will discuss, in turn, the findings related to SD, skewness, kurtosis, and implausible outliers. We compare, where possible, our findings from small-scale refugee surveys to those from the large national DHS surveys (Table 5) [9] and formulate the potential implications of these findings for defining quality assessment criteria. We found that surveys conducted in refugee settings in Uganda tended to have an unusually high SD compared with surveys from other settings, which may be an indication of problematic measurement quality. We, therefore, take a conservative approach in our interpretation and use, in this discussion summary, results for SD, skewness, and kurtosis that exclude surveys from Uganda when comparing to DHS results or proposing plausible ranges. There is a multitude of possible problems leading to inaccurate Hb measurements that can arise in the field, such as excessive squeezing (“milking”) the finger, overfilling of the cuvette, using the first instead of the third drop of capillary blood, the presence of air bubbles in the cuvette, inappropriate cuvette storage, using of expired cuvettes, among many others described in more detail elsewhere [4, 23]. It is therefore of utmost importance to conduct proper training of measurers, implement thorough supervision of the field work, and closely follow all standard procedures and quality assurance steps as described in the Module 3 “Anemia” of the SENS guidelines [5].

Regarding the SD of Hb distributions, we note two important findings. First, SD in WRA tends to be somewhat higher on average than SD in children, with a difference of about 0.08 g/dL. This is remarkably similar to what was found in DHS surveys [9] where SD in WRA was also higher on average by about the same value (0.1 g/dL) (Table 5). This leads us to hypothesize that these differences in SD magnitude between WRA and children are due to inherent properties of Hb distributions in these demographic groups rather than a result of differences in data quality. We also note that SD observed, on average, in DHS data were slightly higher (by about 0.14 g/dL) than those observed in our analysis: 1.58 vs. 1.43 in WRA and 1.48 vs. 1.34 in children, respectively. Rather than attributing this difference to the differences in Hb data quality between DHS and UNHCR surveys, we hypothesize that this difference may be explained by the fact that smaller refugee populations are often homogenous ethnically and have very similar access to food, health services, shelter, water and sanitation, among other factors, and may have slightly lower Hb variability than the Hb variability found in larger, nationally representative populations.

The second important finding for SD is that even after excluding surveys from Uganda, a substantial percentage of both WRA (33%) and child (13%) surveys had SDs exceeding 1.5 g/dL, the upper limit of the plausible 1.1–1.5 g/dL SD range previously proposed by Sullivan et al. [12]. The same phenomenon albeit to a much larger degree is also seen in DHS surveys where SD of Hb distribution exceeded 1.5 g/dL in 71% of surveys among WRA and 46% of surveys among children [9]. This similarity leads us to suggest that the upper plausible range of SD can be raised both for children and for WRA, and more so for WRA since, as described above, WRA tend to have, on average, higher SD than children. We can provisionally suggest the values that approximately correspond to the 90^th^ percentile of SD in our empirically observed distributions excluding Uganda surveys (rounded to the nearest 0.05): 1.55 for children and 1.65 for WRA. Interestingly, the analysis of DHS surveys found a large negative correlation (Pearson’s *r* = -0.68) between the mean and SD of Hb in child data, implying that in populations with higher anemia prevalence, the SD of Hb distributions tends to be markedly larger [9]. We found a similarly high negative correlation between Hb mean and SD in children (excluding Uganda surveys): Spearman’s rho = -0.49 (Pearson’s *r* = -0.43). Unfortunately, the DHS report only reported the mean SD correlation in children and not in WRA [9]. One possible explanation of this phenomenon is that settings with lower resources may be more likely to have both higher anemia (thus lower mean Hb) and lower capacity to produce accurate Hb measurements (thus higher SD of Hb).

For skewness, we note two important phenomena. First, both WRA and child Hb distributions tend to be negatively skewed, and second, WRA distributions tend to be more skewed to the left, on average, than child distributions (mean skewness across all surveys -0.55 and -0.38, respectively). Positive skewness was quite unusual—less than 2% of all child or WRA surveys had skewness above + 0.2. Remarkably, DHS surveys showed the same phenomenon (Table 5)—most of the child and WRA Hb distributions were skewed to the left and WRA distributions were on average more skewed than those in children (− 0.61 vs. − 0.39, respectively).

Regarding kurtosis, we also note two important phenomena. First, both WRA and child Hb distribution across all surveys tend to have positive kurtosis (indicative of relatively large tails and a small body of the distribution), and second, WRA distributions tend to be more positively kurtotic than child distributions (mean kurtosis across all surveys 1.22 vs. 0.55, respectively). Negative kurtosis was minimal—less than 1% of all child or WRA surveys had kurtosis below -0.5. DHS surveys demonstrated the same phenomenon (Table 5)—most of the child and WRA Hb distributions were positive and WRA distributions were, on average, more positively kurtotic than the child ones: 1.25 vs. 0.54 [9]. Such notable consistency in skewness and kurtosis tendencies, in both small-scale refugee SENS surveys and large national DHS surveys, leads us to suggest that these distinctive features of distribution shape are inherent characteristics of child and WRA Hb distributions and not artifacts of low data quality. This consistency also leads to a suggestion for data quality checks based on our empirical evidence: surveys with highly unusual shapes of distributions (defined as skewness above + 0.2 and/or kurtosis below -0.5) may be flagged for further investigation of possible quality problems.

In surveys with available Hb data for both WRA and children, excluding Uganda, SD of Hb were highly correlated (Spearman’s rho = 0.43), suggesting that in surveys with lower quality (more dispersion) of child Hb data WRA data also tends to have a lower quality as indicated by larger dispersion. The correlations of skewness and kurtosis were low, indicating no relationship between the degree of skew or kurtosis in Hb distributions of WRA and children in the same population.

Finally, our data largely agrees with the previously proposed 4.0–18.0 g/dL flagging range for plausible Hb values used in several recent publications, including the DHS report [9, 10, 12]. In our data, only 0.08% of all observations in children and 0.14% of all observations in WRA were outside of this range. Given that only 2.2% of all surveys in children and 3.2% of surveys in WRA had more than 1% of flags defined by this exclusion range, defining > 1% of flags as a quality problem seems logical.

A major strength of this study is the large number of surveys conducted using standard sampling approaches and consistent standard equipment and procedures for data collection. Our data encompasses the period of the last 10 years and, thus, reflects the most recent field practices from a variety of refugee settings in 26 countries. This study, however, has several notable limitations. First and foremost, Hb in SENS surveys is measured via drops of capillary blood using HemoCue 301 devices, which remains a standard practice in field surveys across world regions and survey platforms [5]. Therefore, our findings and suggested quality assessment ranges are not generalizable to Hb data measured by methods other than HemoCue 301 photometry, and even to data measured by HemoCue 301 using venous as opposed to capillary blood. For instance, one recent study showed marked differences in mean, SD, skewness and kurtosis of Hb distributions in data measured by HemoCue in venous versus capillary blood in both children and WRA [10]. Second, the surveys we analyzed were conducted predominantly in populations with high levels of anemia, including more surveys with lower anemia prevalence may produce slightly different results. The distribution of the prevalence of anemia in analyzed surveys in Table 3 shows that over 50% of all surveys in children and close to 25% of all surveys in WRA had prevalence of anemia above 40%. At the same time, in Fig. 2 we demonstrate that there is a substantial negative correlation between Hb mean and SD, especially pronounced in child surveys. Therefore, surveys with low anemia (and therefore higher Hb mean) tend to have lower SD than those with high anemia and low Hb mean. Hence, including more surveys with lower anemia prevalence that would potentially have lower SD could lower overall mean, median and percentile estimates of Hb SD across surveys. Lastly, the current analysis did not include children aged 0–6 months, as this age group is not routinely included in small-scale anthropometric surveys collected in refugee and other humanitarian settings.

## Conclusions

Experts have called for the establishment of postanalytical quality controls for Hb determination—with a specific emphasis on cleaning, adjusting, and analyzing Hb results [1, 3, 5]. From our analyses of population Hb distributions in non-pregnant WRA and children in refugee settings, we observed several intrinsic statistical characteristics that were not necessarily indicative of data quality problems and bear remarkable similarities with the characteristics found in large national DHS surveys. We found that Hb distributions tend to be negatively skewed and positively kurtotic and that distributions in WRA tend to have more left skew and positive kurtosis than child Hb distributions. Moreover, WRA Hb SDs tend to be larger than child Hb SDs, and a considerable proportion of surveys in both groups had SD that exceeded the commonly cited upper acceptable SD value of 1.5 g/dL [12].

Based on our empirical data and on that described in the DHS report [9], we suggest that survey Hb distributions (as determined by the HemoCue 301 system with a single drop of capillary blood) displaying any of the following characteristics be flagged for additional quality investigation: skewness above + 0.2 and/or kurtosis below -0.5, Hb. SD in children aged 6–59 months below 1.1 or above 1.55 g/dL, and Hb SD in non-pregnant women aged 15–49 years below 1.1 and above 1.65 g/dL. The commonly cited minimum and maximum plausible Hb values, 4.0 to 18.0 g/dL, appear to be appropriate [12]. The presence of more than 1% implausible Hb values outside of this range in a given survey may indicate a quality problem. Further investigation is required to determine the reproducibility of our results when Hb is measured using other methods or in blood other than capillary.

## Data Availability

The underlying data that support the findings of this study are available from the United Nations High Commissioner for Refugees, but restrictions apply to the availability of these data, which were used under license for the current study and are not publicly available. Data are, however, available from the authors upon reasonable request and with permission of the United Nations High Commissioner for Refugees.

## Abbreviations

CDC: Centers for Disease Control and Prevention
CI: Confidence interval
DHS: Demographic and Health Surveys
Hb: Hemoglobin
SD: Standard deviation
SENS: Standardized Expanded Nutrition Survey
UNHCR: United Nations High Commissioner for Refugees
WHO: World Health Organization
WRA: Women of reproductive age
